# Cannabidiol Displays Proteomic Similarities to Antipsychotics in Cuprizone-Exposed Human Oligodendrocytic Cell Line MO3.13

**DOI:** 10.3389/fnmol.2021.673144

**Published:** 2021-05-28

**Authors:** Ana Caroline Brambilla Falvella, Bradley Joseph Smith, Licia C. Silva-Costa, Aline G. F. Valença, Fernanda Crunfli, Antonio W. Zuardi, Jaime E. Hallak, José A. Crippa, Valéria de Almeida, Daniel Martins-de-Souza

**Affiliations:** ^1^Laboratory of Neuroproteomics, Department of Biochemistry and Tissue Biology, Institute of Biology, University of Campinas (UNICAMP), Campinas, Brazil; ^2^Department of Neurosciences and Behavior, Faculty of Medicine of Ribeirão Preto, University of São Paulo, Ribeirão Preto, Brazil; ^3^Instituto Nacional de Biomarcadores em Neuropsiquiatria (INBION) Conselho Nacional de Desenvolvimento Científico e Tecnológico, São Paulo, Brazil; ^4^Experimental Medicine Research Cluster (EMRC), University of Campinas, Campinas, Brazil; ^5^D’Or Institute for Research and Education (IDOR), São Paulo, Brazil

**Keywords:** benztropine, haloperidol, clozapine, MO3.13 cell line, phytocannabinoid, proteome, schizophrenia

## Abstract

Cannabidiol, a compound of *Cannabis sativa*, has been proposed as an alternative treatment of schizophrenia. Preclinical and clinical data have suggested that cannabidiol shares more similarity with atypical antipsychotics than typical, both of which are customarily used to manage schizophrenia symptoms. While oligodendrocytes are known to be relevant targets of antipsychotics, the biochemical knowledge in this regard is still limited. Here we evaluated the molecular pathways modulated by cannabidiol compared to the antipsychotics clozapine (atypical) and haloperidol (typical), additionally evaluating the effects of benztropine, a muscarinic receptor antagonist that displays a protective effect in oligodendrocytes and myelination. For this purpose, we employed nano-chromatography coupled with mass spectrometry to investigate the proteomic response to these drugs both in healthy oligodendrocytic cells and in a cuprizone-based toxicity model, using the human oligodendrocyte precursor cell line MO3.13. Cannabidiol shares similarities of biochemical pathways with clozapine and benztropine, in agreement with other studies that indicated an atypical antipsychotic profile. All drugs tested affected metabolic and gene expression pathways and cannabidiol, benztropine, and clozapine modulated cell proliferation and apoptosis when administered after cuprizone-induced toxicity. These general pathways are associated with cuprizone-induced cytotoxicity in MO3.13 cells, indicating a possible proteomic approach when acting against the toxic effects of cuprizone. In conclusion, although modeling oligodendrocytic cytotoxicity with cuprizone does not represent the entirety of the pathophysiology of oligodendrocyte impairments, these results provide insight into the mechanisms associated with the effects of cannabidiol and antipsychotics against cuprizone toxicity, offering new directions of study for myelin-related processes and deficits.

## Introduction

Schizophrenia is a severe and chronic mental disorder that affects over 20 million people worldwide. It is characterized by positive, negative, and cognitive symptoms (Azorin et al., 2014; Owen et al., 2016) which are primarily treated with antipsychotics. These are classified as typical, such as haloperidol, or atypical, including clozapine, the prototype of this drug class (Leucht et al., 2009; van Os and Kapur, 2009) and has since become an indispensable part of treatment in the clinic. Recently, cannabidiol—a compound of *Cannabis sativa—*has been proposed as a new treatment for schizophrenia, since it has been shown to decrease positive symptoms and improve cognitive performance (Leweke et al., 2012; McGuire et al., 2018). Cannabidiol appears to present a pharmacological profile similar to atypical antipsychotics (Gururajan et al., 2011; Zuardi et al., 2012); however, its effects on oligodendrocytes (OLs) are still to be better understood.

OLs are cells present in brain white matter and are responsible for axon myelination, and dysfunction of this process has been implicated in neuronal dysconnectivity (Cassoli et al., 2015; Jørgensen et al., 2016; Vikhreva et al., 2016). This dysconnectivity and abnormalities in white matter have been implicated in the pathophysiology of schizophrenia (Bernard et al., 2015; Orban et al., 2017; Saito et al., 2018). Impairments in oligodendrogenesis and differentiation of oligodendrocyte progenitor cells (OPCs) have also been documented in schizophrenia (Hattori et al., 2014; Santos et al., 2019). Several findings suggest that antipsychotics and cannabidiol affect OLs and myelination, indicating a potential therapeutic effect in schizophrenia and neurodegenerative disorders (Bartzokis et al., 2007, 2009; Zajicek and Apostu, 2011; Rahimi et al., 2015). Thus, the biology of OPCs and mature OLs is an essential focal point to understand the role of these cells in the pathophysiology of—and the development of new treatment for—schizophrenia (de Almeida and Martins-de-Souza, 2018).

Several models exist at various stages of development to comprehend the mechanisms of myelination, demyelination, and remyelination, including the *in vitro* and *in vivo* cuprizone demyelination models (Bénardais et al., 2013; Sachs et al., 2014; Xu et al., 2014, 2019; Taraboletti et al., 2017; de Rosa et al., 2019; Zahednasab et al., 2019). Cuprizone is a copper-chelating agent that inhibits several enzymes that use copper as a cofactor, including cytochrome oxidase, succinyl dehydrogenase, and monoamine oxidase (Venturini, 1973; Petronilli and Zoratti, 1990; Messori et al., 2007). Consequently, this drug induces OL apoptosis, microglial activation, and demyelination in animals (Goldberg et al., 2013; Sachs et al., 2014). Disturbances in energy and protein metabolism have been reported as the molecular mechanisms of cuprizone toxicity, in addition to the resultant increases in oxidative and endoplasmic reticulum stress in OLs (Biancotti et al., 2008; Goldberg et al., 2013; Praet et al., 2014; Taraboletti et al., 2017). Conversely, benztropine is a molecule that enhances OL differentiation and myelination both *in vitro* and *in vivo* (Deshmukh et al., 2013; Ettle et al., 2016; Thompson et al., 2018). This compound is understood to act on immature OLs *via* muscarinic receptor antagonism (Deshmukh et al., 2013; Ettle et al., 2016). However, these receptors’ role and the molecular mechanisms behind the improvements in myelination and OL differentiation are unclear.

Therefore, we hypothesized the ability of cannabidiol to induce protective molecular mechanisms, with similarity to antipsychotics and benztropine in an *in vitro* cuprizone model. Proteomic changes induced by antipsychotics (haloperidol and clozapine), cannabidiol, and benztropine were quantified in oligodendrocytic cells (MO3.13) previously exposed to cuprizone. This study ascertained the degree to which cannabidiol and antipsychotics induce protective molecular mechanisms against cuprizone-mediated cytotoxicity in MO3.13 cells, subsequently comparing these results with benztropine.

## Materials and Methods

### Cell Culture and Treatment

MO3.13 is an immortal oligodendrocytic human-human hybrid cell line that expresses phenotypic characteristics of immature OLs (Buntinx et al., 2003; Iwata et al., 2013), and has been previously used to study oligodendrocyte-like features (Iwata et al., 2013; Brandão-Teles et al., 2017; Jinsmaa et al., 2020). Here, MO3.13 cells were cultured in DMEM (Sigma-Aldrich, St. Louis, MO, United States) supplemented with 10% FBS and penicillin 100 IU/ml, streptomycin 100 μg/ml and grown at 37°C and 5% CO_2_ as described previously (Iwata et al., 2013; Brandão-Teles et al., 2017).

Stocks of cuprizone (28.05 mM; Sigma-Aldrich, St. Louis, MO, United States), haloperidol (53 mM; Cristália), clozapine (25 mM; Cristália), or cannabidiol (25 mM; 99.6% pure with no other cannabinoid, BSPG-Pharm, Sandwich) were prepared in DMSO, and the final concentration of DMSO in the medium was limited to 0.042%. Benztropine (6 mM) was dissolved in ddH_2_O. Saline in DMSO (final concentration of DMSO in medium 0.042%) was used as a control. Based on our pilot experiments and previous studies (Mecha et al., 2012; Bénardais et al., 2013; Deshmukh et al., 2013; Xu et al., 2014; Ettle et al., 2016), MO3.13 cells were seeded in six-well plates at a density of 115,000 cells/well, deprived of FBS overnight, and treated either with 10 μM of cuprizone for 48 h or with 1 μM haloperidol, clozapine, cannabidiol, or benztropine for 24 h. For co-treatment conditions, cells were treated with cuprizone for a total of 48 h; the co-treatment was added during the last 24 h of the treatment, all at the previously mentioned concentrations.

### MTT Assay

For MTT colorimetric assays, cells were seeded in 96-well plates at a density of 7,000 cells/well for 24 h and deprived of FBS overnight. After incubation, the medium was aspirated and replaced with new media containing increasing cuprizone concentrations for 48 h, or haloperidol, clozapine, cannabidiol, or benztropine for 8 h. Subsequently, 0.5 mg/ml of MTT was added to each well and incubated for 2 h at 37°C; ethanol 100% was added, and the absorbance was read at 570 nm on a plate spectrophotometer. Data are expressed as mean ± SEM, and one-way analysis of variance (ANOVA) was used to analyze the statistical significance, followed by Dunnett’s *post-hoc* test (*p* < 0.05).

### Proteome Extraction

After treatment, cells were collected with a scraper and PBS (phosphate-buffered saline; final concentration 1x), and centrifuged at 4°C and 1,200× *g* for 5 min. The pellets were homogenized in a lysis buffer (Tris pH 6.8, 1 M; SDS 20%) with protease inhibitor (final working concentration, 1X; Roche; Mannheim, Germany). Each sample underwent a short run through a polyacrylamide gel to remove buffer salts and other contaminants before peptide digestion was performed *in gel*. One-hunderd microliters of 10 mM dithiothreitol stock (15.4 mg DTT in 10 ml of 50 mM ammonium bicarbonate—Ambic), 100 μl of 55 mM iodoacetamide stock (102 mg IAA in 10 ml of 50 mM Ambic), and 200 μl acetonitrile 100% were added in each sample. Proteins were digested by trypsin for 16–18 h at 37°C and the peptide concentrations were determined by the BSA assay kit (Sigma-Aldrich, St. Louis, MO, United States).

### Tandem Mass Spectrometry

Proteomic samples were separated two-dimensionally and analyzed by a nanoAcquity UPLC M-Class (Waters Corporation, Milford, MA, USA) liquid chromatograph coupled to a Synapt G2-Si mass spectrometer (Waters Corporation, Milford, MA, USA). Each sample was fractionated into five fractions in the first dimension using discontinuous acetonitrile steps (11.4, 14.7, 17.4, 20.7, and 50%) before separation in the second dimension with a continuous acetonitrile gradient from 7% to 40% (v/v) on C18 columns. Peptides were ionized by electrospray ionization in positive mode and [Glu 1]-Fibrinopeptide B was used as the lock-mass. MS/MS analyses were performed in data-independent acquisition (DIA) mode using UDMS^E^ after manually creating an ion selection method profile for each sample fraction using DriftScape (version 2.9).

### Data Processing and Protein Identification

MS/MS spectra were aligned and analyzed with Progenesis^®^ QI for Proteomics (version 3.1) with Apex3D, peptide 3D, and ion accounting informatics (Waters). Proteins were identified using the reviewed Uniprot *Homo sapiens* database (obtained 12/2020) and quantified using the default Top3 method. The following parameters were used in the identification of peptides/proteins: digestion by trypsin with a maximum of one missed cleavage; maximum protein mass of 600 kDa; false discovery rate (FDR) less than 1% (as calculated by a reverse sequence database generated on-the-fly by Progenesis); at least two fragments per peptide, one peptide per protein, and five or more fragments per protein; at least one unique peptide per protein for quantitation; and a mass error ≤20 ppm. Proteins were considered differentially expressed when the one-way analysis of variance (ANOVA) returned a *p*-value < 0.05.

### *In silico* Analyses

Using proteins considered to be differentially expressed, affected biological processes and biochemical pathways were analyzed using Reactome (Fabregat et al., 2018), David bioinformatics database (Huang et al., 2009a,b), and Proteomaps (Liebermeister et al., 2014). Biological processes and protein networks were analyzed using Metascape (Zhou et al., 2019), Cytoscape version 3.8.2 (Bindea et al., 2009; Lotia et al., 2013; Franz et al., 2016; Szklarczyk et al., 2017), and ClustVis (Metsalu and Vilo, 2015).

## Results

### MTT Assay

Cuprizone-induced toxicity was measured in the human oligodendrocytic cell line MO3.13 using increasing cuprizone concentrations for 48 h *via* MTT assay. All cells treated with cuprizone displayed a loss of viability after 48 h (Supplementary Figure 1A). The concentrations used in previous studies for these antipsychotics, cannabidiol, and benztropine (Mecha et al., 2012; Deshmukh et al., 2013; Xu et al., 2014) were confirmed to not be cytotoxic to the MO3.13 cell line under this experiment’s conditions. This procedure was achieved by MTT assay, confirming that the cytotoxic cutoff for these compounds at 8 h of incubation is sufficiently above these concentrations. Viability dropped to a significant degree after 75 μM clozapine, 50 μM haloperidol, 4 μM cannabidiol, and 100 μM benztropine (Supplementary Figures 1B–E). The previously published concentration of 10 μM cuprizone induced a cytotoxic effect in these cells and concentrations of 1 μM for antipsychotics, cannabidiol, and benztropine (Mecha et al., 2012; Bénardais et al., 2013; Deshmukh et al., 2013; Xu et al., 2014; Taraboletti et al., 2017) were not cytotoxic and therefore used during treatment, and co-treatment assays.

### Proteomic Analyses

#### Proteomic Signature of MO3.13 Cells

Although the MO3.13 cell line does not exhibit the main features of myelinating OLs nor those of late-stage OPCs that could differentiate into mature OLs, these cells can be useful for the investigations of early stages of OPCs. We previously showed that the MO3.13 cell line submitted to a differentiation protocol presents an increase in the marker PLP, suggesting its OPC-like features (Seabra et al., 2019, 2020). Here, we compared the whole proteome identified in our experiments with the databanks for OLs and OPCs (Zhang et al., 2019), whereupon we found several protein markers in our data, including RAB2A, TUBB, NPM1, LDHB, MAP1A, NACA, DHCR7, TMEFF2, BIN1, NAP1L1, MARCKSL1, EIF3E, DHCR24, RPS2, LIMA1, GSN, SCD, EEF1B2, TUBB3, EEF2, PGRMC1, NME2, ACAT2, MAP2, EIF3L, RPL13A, SNX1, CNP, MIF, HIP1, FDPS, GAP43, CDK18, PDGFRA, MICAL1, RPL31, CDH13, RPS23, TM7SF2, and TUBB4A. Together, these data reinforce that MO3.13 cells can be a useful approach to investigate the molecular pathways for an early stage of OPCs. In line with these data, other studies have also shown oligodendrocytic markers in MO3.13 cells (McLaurin et al., 1995; Buntinx et al., 2003).

#### Cannabidiol, Antipsychotics, and Benztropine Individually

**Figure 1:**
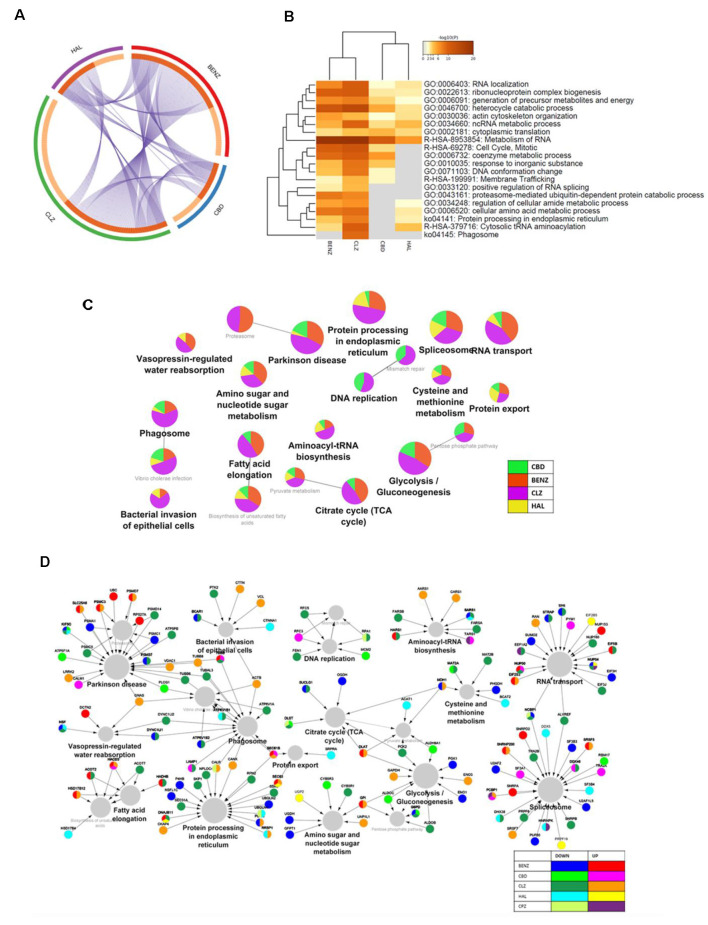
**(A)** Chord diagram. **(B)** Heatmap of enriched pathways (Metascape). **(C)** Network of pathways affected by cannabidiol (CBD), benztropine (BENZ), clozapine (CLZ), and haloperidol (HAL) treatments, according to proteomic analysis, showing the percentage of visible genes of each term or pathway. **(D)** Network of pathways and proteins affected by cannabidiol, benztropine, clozapine, and haloperidol treatments, and cuprizone (CPZ) insult, according to proteomic analysis, showing hub genes that are shared in the enriched KEGG pathways. The gene ontology interaction network was obtained by functional enrichment analysis using the ClueGO plug-in in Cytoscape. All terms were selected by significance (*p* < 0.05) following a Benjamini-Hochberg correction.

A total of 1,890 proteins were identified and quantified. When compared to vehicle, differentially expressed proteins were found in each condition: cannabidiol, 93; haloperidol, 97; clozapine, 278; and benztropine, 191 (see Supplementary Table 1). The most extensive overlap of proteins and pathways affected in the same direction was observed between clozapine and benztropine, while cannabidiol shared a higher overlap of pathways with clozapine and benztropine than with haloperidol (Figures 1A,B,D). Regarding biological processes, all drugs caused changes in RNA metabolism, cytoplasmic translation, ncRNA metabolic process, actin cytoskeleton organization, heterocycle catabolic process, generation of precursor metabolites and energy, ribonucleoprotein complex biogenesis, and RNA localization (Figure 1B). Treatment with benztropine, cannabidiol, or antipsychotics additionally modulated proteins related to various metabolic processes (Figures 1C,D and Supplementary Figures 2B–E) and antioxidant defense (Supplementary Table 2).

#### Proteome of MO3.13 Cells Treated With Cuprizone

**Figure 2:**
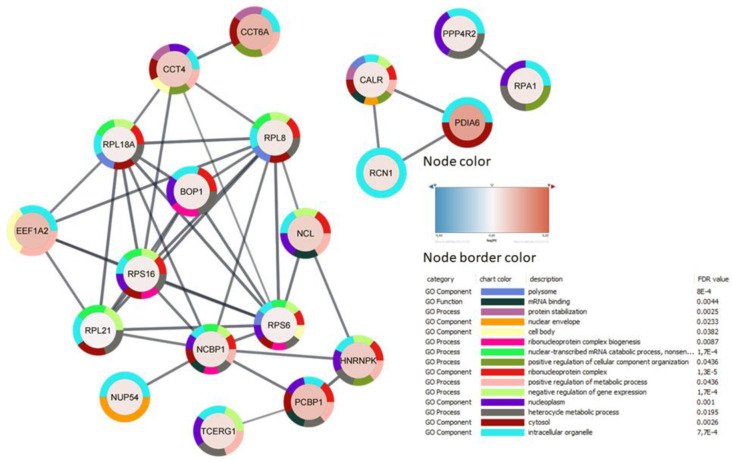
Protein-protein interaction of 44 genes affected by cuprizone treatment, analyzed with STRING in Cytoscape. Proteins without interactions within the network were omitted. Nodes represent proteins, their colors indicate the fold-change in the expressed pattern after cuprizone treatment, and lines represent interactions between them. Node border colors indicate the top 15 gene ontology terms.

A total of 1,889 proteins were identified and quantified, 44 of which were differentially expressed in MO3.13 cells in response to cuprizone (see Supplementary Table 1). In line with the literature (Werner et al., 2010; Taraboletti et al., 2017; Szilagyi et al., 2020) here we also identified proteins related to metabolism, including L-lactate dehydrogenase A chain (LDHA), polyunsaturated fatty acid 5-lipoxygenase (ALOX5), and dihydrolipoyl lysine-residue succinyltransferase component of 2-oxoglutarate dehydrogenase (DLST; Supplementary Table 2). Cuprizone was also found to modulate proteins related to amino acid metabolism and genetic processes, including translational, spliceosome processes (FDR: 0.0064, String-DB), and RNA splicing (Figure 2 and Supplementary Figure 2A).

#### Proteomic Changes in Co-treated MO3.13 Cells

**Figure 3:**
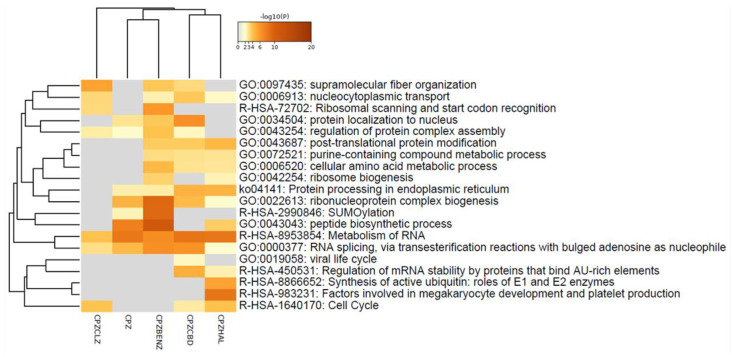
Heatmap of enriched pathways of MO3.13 cells insulted with cuprizone (CPZ) or co-treated with cuprizone and haloperidol (CPZHAL), cannabidiol (CPZCBD), clozapine (CPZCLZ), or benztropine (CPZBENZ). Created in Metascape.

**Figure 4:**
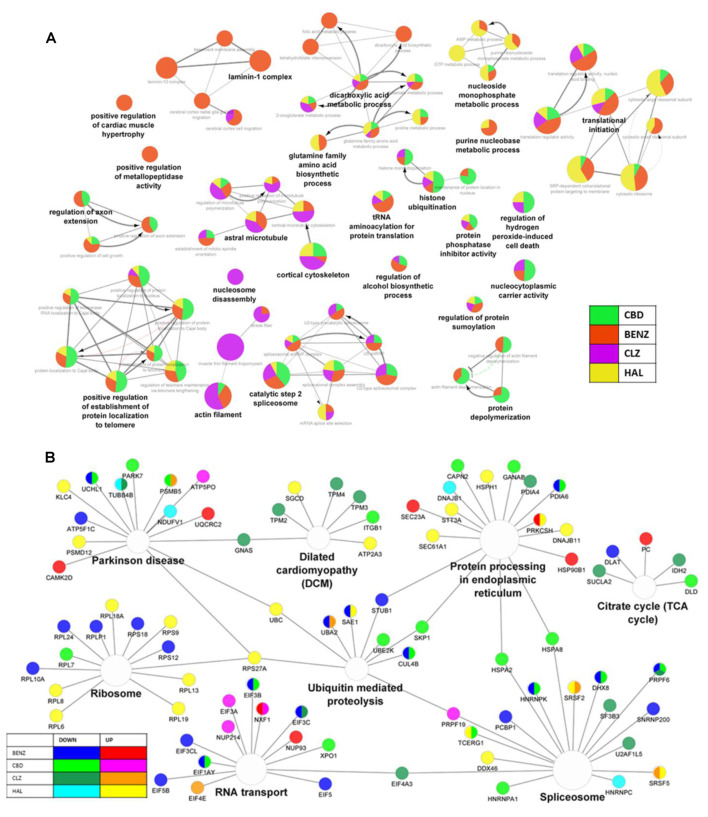
**(A)** The network of gene ontology terms, affected by cannabidiol, benztropine, clozapine, and haloperidol co-treatments compared to cuprizone, according to proteomic analysis, showing the percentage of visible genes of a term or pathway. **(B)** Network of pathways affected by cannabidiol, benztropine, clozapine, and haloperidol co-treatments, according to proteomic analysis, showing the hub genes shared between the enriched KEGG pathways. The gene ontology interaction network was obtained by functional enrichment analysis using the ClueGO plug-in in Cytoscape. All terms were selected by significance (*p* < 0.05) following a Benjamini–Hochberg correction.

A total of 1890 and 1889 proteins were identified and quantified for CPZCBD and CPZCLZ and CPZHAL and CPZBENZ, respectively. When compared to cells insulted with cuprizone, differentially expressed proteins were found in co-treatment with cannabidiol (CPZCBD, 89), haloperidol (CPZHAL, 92), clozapine (CPZCLZ, 75), and benztropine (CPZBENZ, 150; see Supplementary Table 1). Unlike the pathway clustering organization observed when drugs were administered alone (Figure 1B), the comparison of co-treatments indicated that cannabidiol shares more proteins that are dysregulated in the same direction with benztropine than with haloperidol or clozapine (Figures 4A,B). All co-treatments caused changes [−log_10_ (P) from 20 to 2] in proteins related to RNA metabolism and RNA splicing (Figures 3, 4B). For details regarding proteins and genes, see Figure 4B and Supplementary Figure 3. Moreover, these co-treatments modulated proteins seen in cuprizone insult, including protein disulfide-isomerase, heterogeneous nuclear ribonucleoprotein K, T-complex protein 1 subunit zeta, and protein LYRIC, which were all modulated in both cannabidiol and benztropine co-treatments, with both antipsychotics also modulating myeloid-associated differentiation marker (MYADM).

## Discussion

Comparing the effects of cannabidiol and antipsychotics with the putative protective function of benztropine on the MO3.13 proteome, we found that all drugs modulated proteins related to metabolism, antioxidant system, and RNA splicing. Here, we found changes in the constituent proteins of several molecular mechanisms and biological processes in oligodendrocytic cells resulting from benztropine administration, which might be associated with its ability to induce myelination and OPC differentiation. We then compared these results with MO3.13 cells treated with cannabidiol and two antipsychotics (haloperidol and clozapine) in terms of their hypothesized ability to induce protective molecular mechanisms.

First, we showed that benztropine modulated proteins related to energy, cholesterol, and fatty acid metabolism, which might be associated with its mechanisms of inducing cholesterol biogenesis in OLs and myelination (Ettle et al., 2016; Hubler et al., 2018). Cannabidiol and antipsychotics are previously known to modulate proteins related to catabolic processes. Studies have demonstrated that cannabidiol can attenuate cell damage by promoting mitochondrial function and biogenesis, thus modulating glucose metabolism in neurons (Sun et al., 2017). The observation that cannabidiol also alters metabolic pathways in MO3.13 cells supports the previous studies and helps validate these findings. Clozapine treatment also modulated proteins related to energy and lipid metabolism. Regarding haloperidol, its administration has been associated with changes in proteins related to carbohydrates and lipid metabolism (Brandão-Teles et al., 2019), similar to our results; however, it inhibits cholesterol biosynthesis in cell culture (Sánchez-Wandelmer et al., 2010). The protective effects of these drugs on MO3.13 cells can be attributed to a modulation of metabolism, similar to benztropine. The result that benztropine modulates proteins related to energy metabolism, as cannabidiol, haloperidol, and clozapine do, suggests that this may be a shared mechanism of action for protective effects.

Our results also reported that benztropine modulated a protein related to antioxidant defense. The antioxidant effects of benztropine are well known in neuronal cells (Cerles et al., 2019). Nonetheless, we found that benztropine downregulated disulfide-isomerase A3, which attenuates oxidative and endoplasmic reticulum stress (Yoo et al., 2019). We found that clozapine modulated the protein superoxide dismutase, related to antioxidant defense, and both cannabidiol and haloperidol modulated proteins related to selenocysteine synthesis and selenoamino acid metabolism. While haloperidol has been suggested to induce oxidative stress in rat brains (Andreazza et al., 2015), clozapine has been described to promote an antioxidant effect (Hendouei et al., 2018). Similarly, cannabidiol has been observed to prevent hydrogen peroxide-induced apoptosis in nucleus pulposus cells and decrease oxidative species in OPCs (Mecha et al., 2012; Chen et al., 2016), suggesting a possible cause of the differences in the side effect profiles of antipsychotics and cannabidiol.

After comparing the individual effects of cannabidiol, antipsychotics, and benztropine, we investigated these drugs’ molecular mechanisms against cuprizone-mediated toxicity in MO3.13 cells. Cuprizone insult resulted in changes in proteins related to metabolism and genetic processes, both of which were also modulated by benztropine treatment. More specifically, benztropine increased some proteins related to fatty acid elongation and decreased proteins related to energy metabolism, as well as both up-and down-regulating proteins related to the spliceosome and RNA transport (Figure 1D). We identified bidirectional modulations in apoptosis and cell proliferation proteins in cannabidiol, clozapine, and haloperidol co-treatments, which were mostly downregulated in response to cuprizone insult. Cannabidiol and benztropine co-treatments increased heterogeneous nuclear ribonucleoprotein K after cuprizone-induced downregulation, a protein which regulates proliferation and apoptosis (Chen et al., 2017). Cannabidiol, benztropine, and clozapine co-treatments also decreased the protein LYRIC, an activator of the transcription factor NF-κB that affects neuronal plasticity, neurodegeneration, and neuronal development (O’Neill and Kaltschmidt, 1997) and which was found increased after cuprizone insult. Lastly, clozapine increased DnaJ homolog subfamily A member 3, a modulator of apoptotic signal transduction that was decreased after cuprizone insult. Though there is significant overlap between clozapine, benztropine, and cannabidiol (Figures 1, 3, 4), each compound seems to affect OL proliferation and apoptosis in distinct ways; these compounds must be tested further regarding their potential to promote cell proliferation.

Regarding energy and lipid metabolism, cuprizone decreases glucose utilization (Taraboletti et al., 2017; Kim et al., 2019), affects NAD+ metabolism, and blocks glycolytic pathways (Taraboletti et al., 2017). In our study, cuprizone appeared to affect metabolic proteins and may impair ATP production, and affect the antioxidant system. Benztropine co-treatment induced changes in metabolic processes, in which modulated proteins were most specifically related to energy and amino acid metabolism, additionally downregulating proteins related to lipid metabolism (Figure 4B). These data, therefore, indicate benztropine’s potential to attenuate the metabolic disturbances seen in the cuprizone model. All co-treatments modulated varying proteins involved in energy and lipid metabolism, and in particular, all drugs increased the expression of aspartate aminotransferase, related to NAD redox balance, which was altered during cuprizone administration (Taraboletti et al., 2017). However, disulfide-isomerase A6, a redox activity regulator which was upregulated in cuprizone insult, was downregulated only by cannabidiol and benztropine co-treatments. Cannabidiol co-treatment modulated dihydrolipoyl dehydrogenase and ATP synthase subunit O, which are related to ATP production. Haloperidol co-treatment also modulated proteins related to energy metabolism, including ATP-dependent 6-phosphofructokinase and NADH dehydrogenase flavoprotein 1. Since clozapine did not significantly modulate biological pathways deregulated by cuprizone, it is interesting that in an *in vivo* cuprizone model, clozapine treatment could not prevent demyelination during cuprizone administration; however, it did enhance functional support after demyelination (Templeton et al., 2019). Here, we showed that clozapine instead modulates some critical proteins related to energy metabolism, such as succinate-CoA ligase and isocitrate dehydrogenase. Thus, we demonstrated that antipsychotics modulate proteins related to metabolism and, similarly, cannabidiol and benztropine modulate this pathway and the antioxidant system throughout cuprizone insult, suggesting a potential ability to prevent demyelination events.

Last, our data also show that cuprizone modulated proteins related to amino acid metabolism and genetic processes, including translation and spliceosome processes. Amino acid perturbations and deregulations in the antioxidant system have already been documented in mice exposed to cuprizone (Goldberg et al., 2013; Taraboletti et al., 2017). Our results agree with the hypothesis that cuprizone can affect amino acid metabolism, including selenoamino acid metabolism. All co-treatments downregulated some proteins related to spliceosomes and RNA metabolism (Figure 4B); and post-translational modification processes were modulated by benztropine, cannabidiol, and haloperidol. In particular, RNA splicing pathways, downregulated after cuprizone insult, showed higher expression of some constituent proteins during the antipsychotic co-treatments (Figures 1C, 4B). Benztropine, however, decreased poly (rC)-binding protein 1, which also participates in RNA splicing, and cannabidiol decreased the levels of threonine-tRNA ligase 1, a protein that participates in protein biosynthesis. It is worth noting that both cannabidiol and benztropine decreased heterogeneous nuclear ribonucleoprotein K, which is a protein related to spliceosome function that was found upregulated after cuprizone insult. Though each co-treatment affected these pathways differently, a more developed understanding of how these pathways are affected by cannabidiol, antipsychotics, and benztropine is essential to help determine how OLs regenerate after toxic effects like those induced by cuprizone, and how schizophrenia and other demyelinating disorders can be better treated.

This study, of course, has its limitations. First, the MO3.13 cell line is not able to properly model myelination and remyelination processes since the cells do not differentiate into myelinating or mature OLs and are not able to myelinate axons (De Kleijn et al., 2019). Second, the cell line is not suitable as a model for the multifactorial disease since it does not carry any genotypic or phenotypic features of patients. Nonetheless, this oligodendrocytic cell line has been previously used to study the early stages of OPC features (Iwata et al., 2013; Brandão-Teles et al., 2017; Jinsmaa et al., 2020) and we previously showed that MO3.13 cells submitted to a short maturation protocol exhibit an increase in PLP, highlighting the utility of these cells to investigate molecular pathways related to the early stages of OPCs (Seabra et al., 2019, 2020). Third, the proteomic analyses performed herein point to several altered pathways and biological processes; but a validation of these findings using other approaches is fundamental to confirm the effects on the functionality of these, and other, cells. In conclusion, our study provides evidence that cannabidiol can modulate signaling pathways similarly to clozapine and benztropine, depicting the biochemical mechanisms behind such modulations. After the cuprizone-induced insult, cannabidiol exhibited similarities with benztropine, clozapine, and haloperidol. Cannabidiol and antipsychotics mainly affect metabolism, cell proliferation, apoptosis, cell redox-homeostasis, and genetic factors. There is an overlap in the pathways affected by cannabidiol, benztropine, and antipsychotics. Since this overlap includes pathways that are documented to be related to protective effects in OLs, we suggest that cannabidiol is indeed a potential protective compound against cuprizone insult in oligodendrocytic cells, which might be related to the demyelination process, a hypothesis that must be confirmed using *in vivo* and more complex *in vitro* models.

## Data Availability Statement

The mass spectrometry proteomics data have been deposited to the ProteomeXchange Consortium via the PRIDE partner repository with the dataset identifier PXD028419.

## Author Contributions

ACBF conducted the experimental part, analyzed data, and wrote the manuscript. BS helped with data analysis, manuscript revision, and English review. LS-C helped with data analysis and figures. AV and FC performed validation experiments. DM-d-S supervised the whole process and provided funding. DM-d-S and VA designed and conceived the study and edited the final version of the manuscript. AZ, JH, and JC provided cannabidiol for experiments, and contributed to manuscript revision. All authors contributed to the article and approved the submitted version.

## Conflict of Interest

JC is a member of the International Advisory Board of the Australian Centre for Cannabinoid Clinical and Research Excellence (ACRE)—National Health and Medical Research Council (NHMRC). JC and JH have received travel support to attend scientific meetings and personal consultation fees from BSPG-Pharm. JC, JH, and AZ are co-inventors of the patent “Fluorinated CBD compounds, compositions and uses thereof. Pub. No. WO/2014/108899. International Application No. PCT/IL2014/050023,” Def. US number Reg. 62193296; July 29, 2015; INPI on August 19, 2015 (BR1120150164927; Mechoulam R, Zuardi AW, Kapczinski F, Hallak JEC, Guimarães FS, Crippa JAS, Breuer A). Universidade de São Paulo (USP) has licensed this patent to Phytecs Pharm (USP Resolution No. 15.1.130002.1.1) and has an agreement with Prati-Donaduzzi to “develop a pharmaceutical product containing synthetic CBD and prove its safety and therapeutic efficacy in the treatment of epilepsy, schizophrenia, Parkinson’s disease, and anxiety disorders.” JC, JH, and AZ are co-inventors of the patent “Cannabinoid-containing oral pharmaceutical composition, method for preparing and using same,” INPI on September 16, 2016 (BR 112018005423-2). The remaining authors declare that the research was conducted in the absence of any commercial or financial relationships that could be construed as a potential conflict of interest.

## Supplementary Material

SUPPLEMENTARY FIGURE 1 A MTT assay was used to measure the percentage of live MO3.13 cells after 48 h and 8 h in growth medium (DMEM), Vehicle (for cuprizone: DMSO, 0.3%; haloperidol and cannabidiol: DMSO, 0.3%; clozapine: DMSO, 0.4%; benztropine: water), and increasing concentrations of cuprizone for 48 h **(A)**, haloperidol **(B)**, clozapine **(C)**, cannabidiol **(D)**, or benztropine **(E)** for 8 h (**P* ≤ 0.05; ****P* < 0.001; *****P* < 0.0001; *n* = 6 wells/condition).

SUPPLEMENTARY FIGURE 2 Bar graph of enriched biochemical pathways in MO3.13 cells treated with cuprizone 10 μM, 48 h **(A)**, haloperidol **(B)**, clozapine **(C)**, cannabidiol **(D)**, and benztropine **(E)**, colored by *p*-value, created in Metascape. Colored lines represent *p*-values, which range between 10^−20^ and 10^−2^.

SUPPLEMENTARY FIGURE 3 Network of pathways affected by cannabidiol, benztropine, clozapine, and haloperidol co-treatments, according to proteomic analysis, showing the percentage of visible genes of a term or pathway and the hub genes that enriched KEGG pathways. The gene ontology interaction network was obtained by functional enrichment analysis using the ClueGO plug-in in Cytoscape. All terms were selected by significance (*p* < 0.05) following a Benjamini-Hochberg correction.

SUPPLEMENTARY TABLE 1 Proteins differentially regulated by treatments.

SUPPLEMENTARY TABLE 2 Pathways associated with proteome changes induced by treatments.
