# Corrigendum: Cannabidiol Displays Proteomic Similarities to Antipsychotics in Cuprizone-Exposed Human Oligodendrocytic Cell Line MO3.13

**DOI:** 10.3389/fnmol.2021.814907

**Published:** 2021-11-30

**Authors:** Ana Caroline Brambilla Falvella, Bradley Joseph Smith, Licia C. Silva-Costa, Aline G. F. Valença, Fernanda Crunfli, Antonio W. Zuardi, Jaime E. Hallak, José A. Crippa, Valéria de Almeida, Daniel Martins-de-Souza

**Affiliations:** ^1^Laboratory of Neuroproteomics, Department of Biochemistry and Tissue Biology, Institute of Biology, University of Campinas (UNICAMP), Campinas, Brazil; ^2^Department of Neurosciences and Behavior, Faculty of Medicine of Ribeirão Preto, University of São Paulo, São Paulo, Brazil; ^3^Instituto Nacional de Biomarcadores em Neuropsiquiatria (INBION) Conselho Nacional de Desenvolvimento Científico e Tecnológico, São Paulo, Brazil; ^4^Experimental Medicine Research Cluster (EMRC), University of Campinas, Campinas, Brazil; ^5^D'Or Institute for Research and Education (IDOR), São Paulo, Brazil

**Keywords:** benztropine, haloperidol, clozapine, MO3.13 cell line, phytocannabinoid, proteome, schizophrenia

In the original article, information from the Data Availability statement was mistakenly omitted. The corrected Data Availability Statement is below.

The mass spectrometry proteomics data have been deposited to the ProteomeXchange Consortium via the PRIDE partner repository with the dataset identifier PXD028419.

The authors apologize for the error and state that this does not change the scientific conclusions of the article in any way. The original article has been updated.

## Publisher's Note

All claims expressed in this article are solely those of the authors and do not necessarily represent those of their affiliated organizations, or those of the publisher, the editors and the reviewers. Any product that may be evaluated in this article, or claim that may be made by its manufacturer, is not guaranteed or endorsed by the publisher.

