# Supplementary figures and images for: Cannabidiol Displays Proteomic Similarities to Antipsychotics in Cuprizone-Exposed Human Oligodendrocytic Cell Line MO3.13
Source: Front Mol Neurosci. 2021 May 28;14:673144. doi: 10.3389/fnmol.2021.673144 (PMC8193732; doi:10.3389/fnmol.2021.673144)

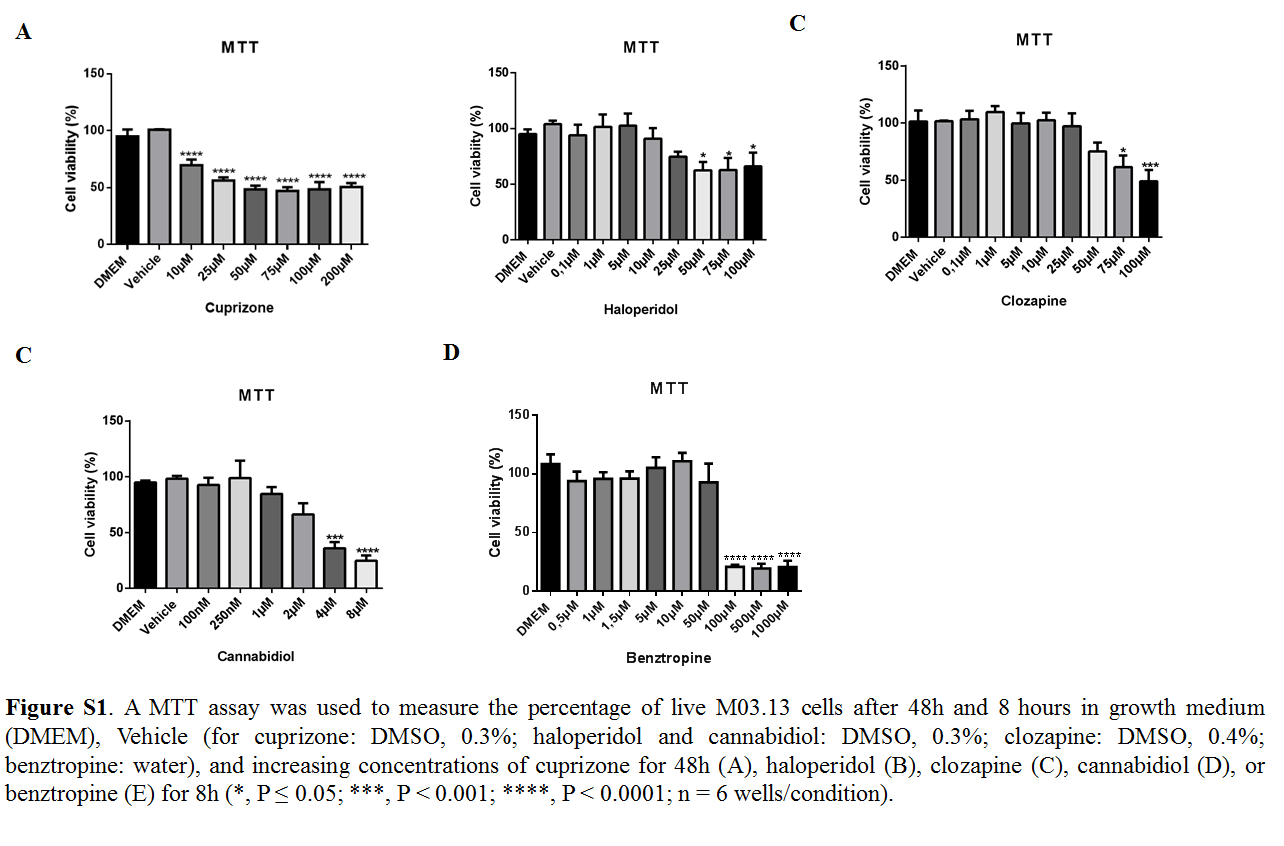

Supplement: SUPPLEMENTARY FIGURE 1 — A MTT assay was used to measure the percentage of live MO3.13 cells after 48 h and 8 h in growth medium (DMEM), Vehicle (for cuprizone: DMSO, 0.3%; haloperidol and cannabidiol: DMSO, 0.3%; clozapine: DMSO, 0.4%; benztropine: water), and increasing concentrations of cuprizone for 48 h (A), haloperidol (B), clozapine (C), cannabidiol (D), or benztropine (E) for 8 h (*P ≤ 0.05; ***P < 0.001; ****P < 0.0001; n = 6 wells/condition). [file Image_1.TIF]

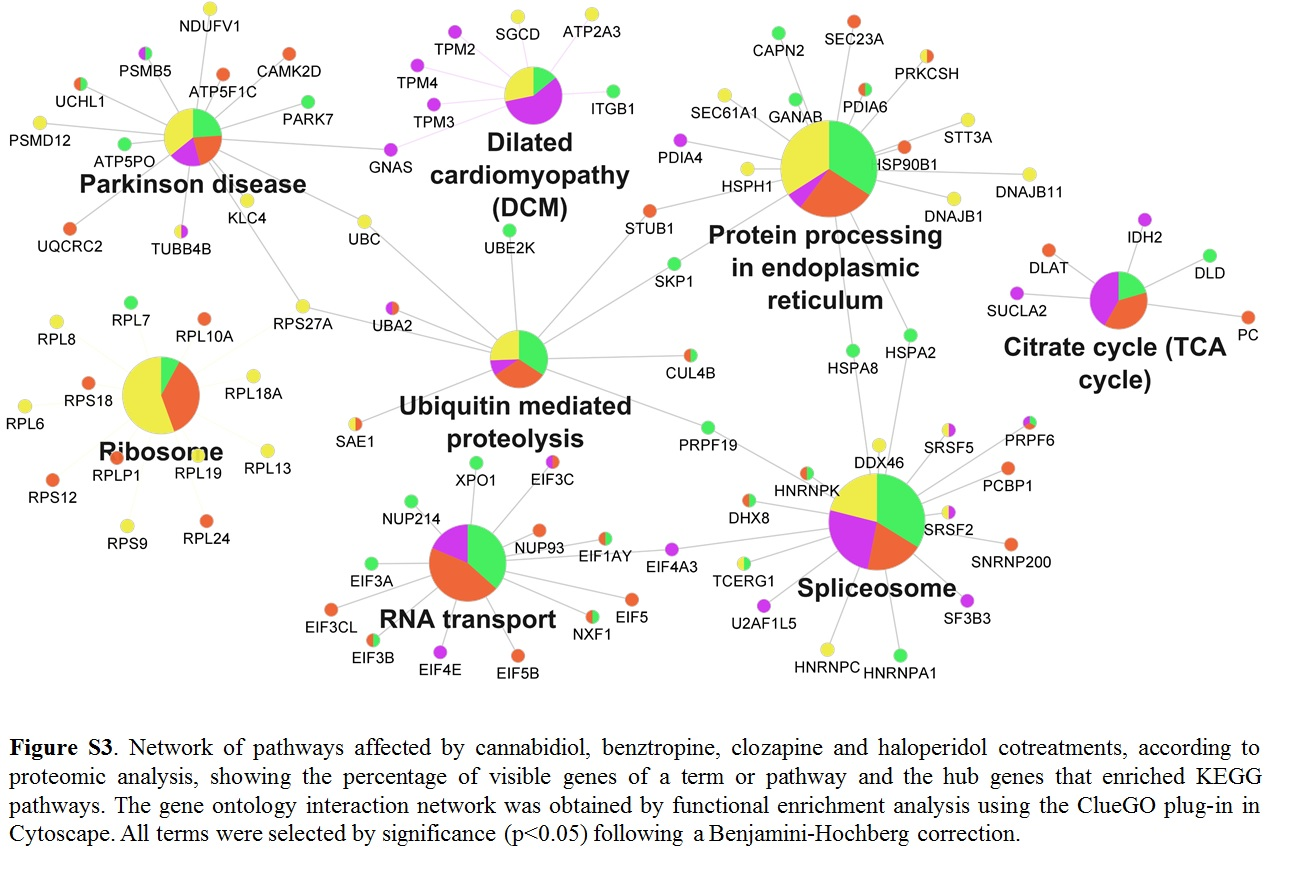

Supplement: SUPPLEMENTARY FIGURE 3 — Network of pathways affected by cannabidiol, benztropine, clozapine, and haloperidol co-treatments, according to proteomic analysis, showing the percentage of visible genes of a term or pathway and the hub genes that enriched KEGG pathways. The gene ontology interaction network was obtained by functional enrichment analysis using the ClueGO plug-in in Cytoscape. All terms were selected by significance (p < 0.05) following a Benjamini-Hochberg correction. [file Image_3.tif]
