# Supplementary material for: Cannabidiol Displays Proteomic Similarities to Antipsychotics in Cuprizone-Exposed Human Oligodendrocytic Cell Line MO3.13
Source: Front Mol Neurosci. 2021 May 28;14:673144. doi: 10.3389/fnmol.2021.673144 (PMC8193732; doi:10.3389/fnmol.2021.673144)
Supplement: SUPPLEMENTARY FIGURE 2 — Bar graph of enriched biochemical pathways in MO3.13 cells treated with cuprizone 10 μM, 48 h (A), haloperidol (B), clozapine (C), cannabidiol (D), and benztropine (E), colored by p-value, created in Metascape. Colored lines represent p-values, which range between 10−20 and 10−2. [file Image_2.pdf]

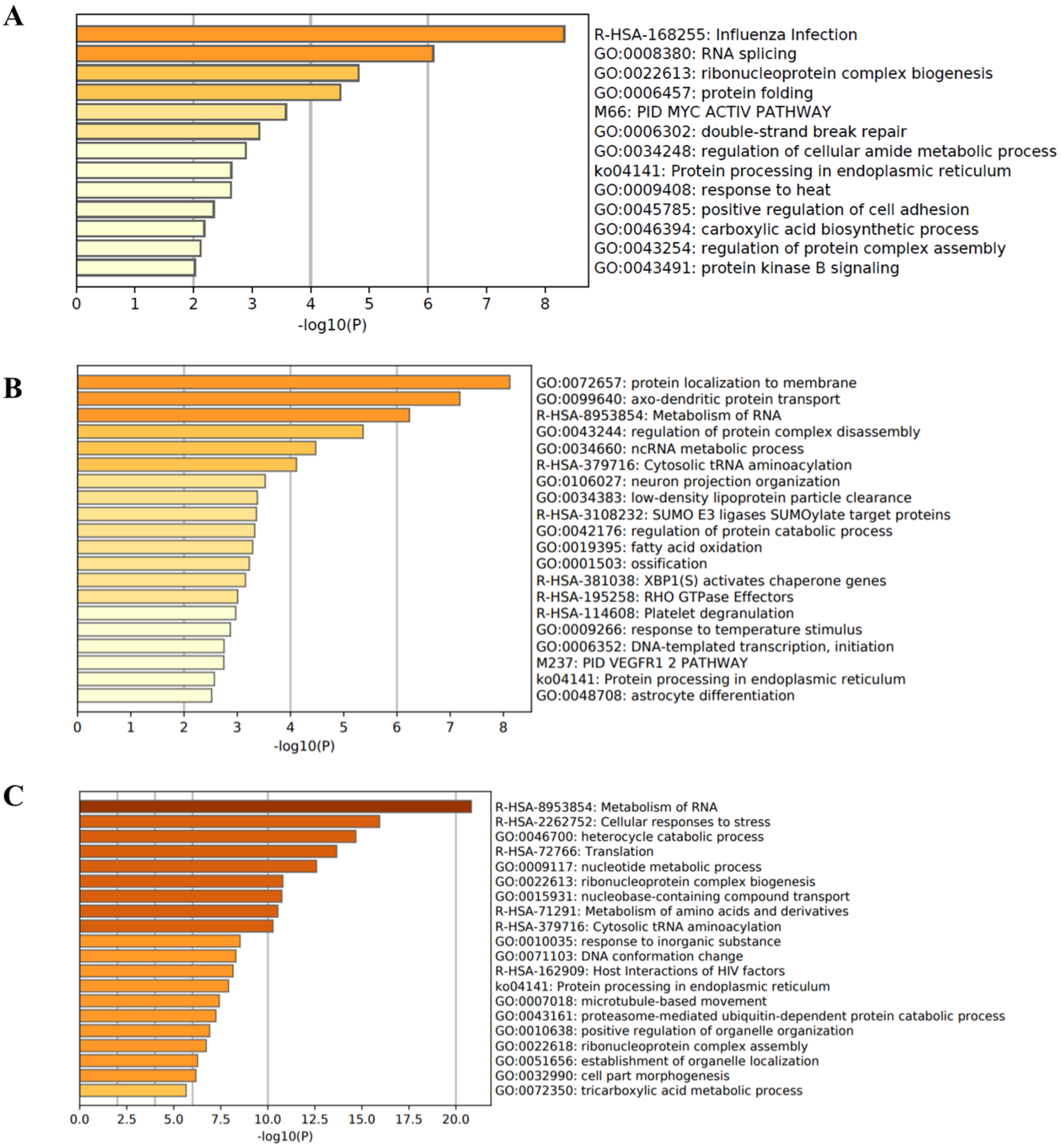
A
R-HSA-168255: Influenza Infection
GO:0008380: RNA splicing
GO:0022613: ribonucleoprotein complex biogenesis
GO:0006457: protein folding
M66: PID MYC ACTIV PATHWAY
GO:0006302: double-strand break repair
GO:0034248: regulation of cellular amide metabolic process
ko04141: Protein processing in endoplasmic reticulum
GO:0009408: response to heat
GO:0045785: positive regulation of cell adhesion
GO:0046394: carboxylic acid biosynthetic process
GO:0043254: regulation of protein complex assembly
GO:0043491: protein kinase B signaling
0 1 2 3 4 5 6 7 8
-log10(P)
B
GO:0072657: protein localization to membrane
GO:0099640: axo-dendritic protein transport
R-HSA-8953854: Metabolism of RNA
GO:0043244: regulation of protein complex disassembly
GO:0034660: ncRNA metabolic process
R-HSA-379716: Cytosolic tRNA aminoacylation
GO:0106027: neuron projection organization
GO:0034383: low-density lipoprotein particle clearance
R-HSA-3108232: SUMO E3 ligases SUMOylate target proteins
GO:0042176: regulation of protein catabolic process
GO:0019395: fatty acid oxidation
GO:0001503: ossification
R-HSA-381038: XBP1(S) activates chaperone genes
R-HSA-195258: RHO GTPase Effectors
R-HSA-114608: Platelet degranulation
GO:0009266: response to temperature stimulus
GO:0006352: DNA-templated transcription, initiation
M237: PID VEGFR1 2 PATHWAY
ko04141: Protein processing in endoplasmic reticulum
GO:0048708: astrocyte differentiation
0 1 2 3 4 5 6 7 8
-log10(P)
C
R-HSA-8953854: Metabolism of RNA
R-HSA-2262752: Cellular responses to stress
GO:0046700: heterocycle catabolic process
R-HSA-72766: Translation
GO:0009117: nucleotide metabolic process
GO:0022613: ribonucleoprotein complex biogenesis
GO:0015931: nucleobase-containing compound transport
R-HSA-71291: Metabolism of amino acids and derivatives
R-HSA-379716: Cytosolic tRNA aminoacylation
GO:0010035: response to inorganic substance
GO:0071103: DNA conformation change
R-HSA-162909: Host Interactions of HIV factors
ko04141: Protein processing in endoplasmic reticulum
GO:0007018: microtubule-based movement
GO:0043161: proteasome-mediated ubiquitin-dependent protein catabolic process
GO:0010638: positive regulation of organelle organization
GO:0022618: ribonucleoprotein complex assembly
GO:0051656: establishment of organelle localization
GO:0032990: cell part morphogenesis
GO:0072350: tricarboxylic acid metabolic process
0.0 2.5 5.0 7.5 10.0 12.5 15.0 17.5 20.0
-log10(P)

D

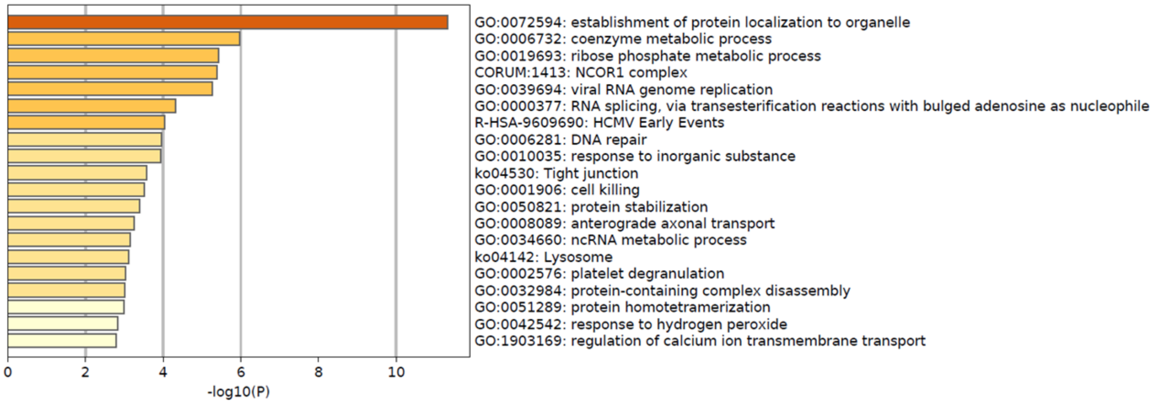

E

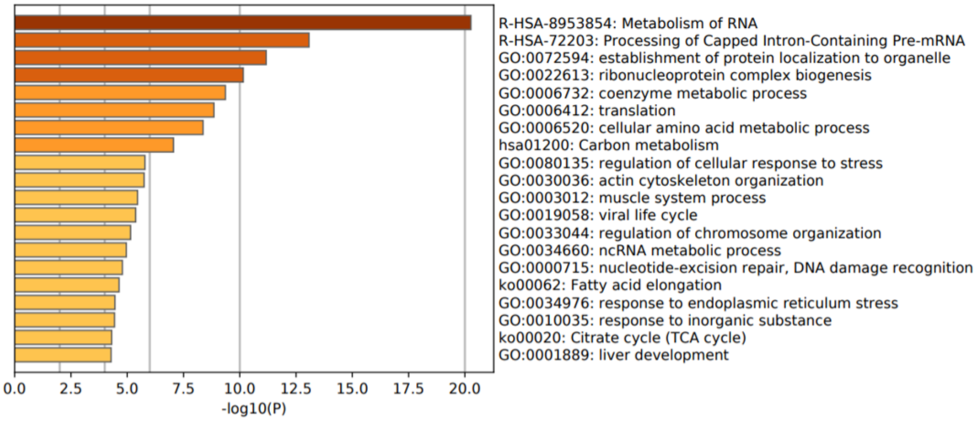

Figure S2. Bar graph of enriched biochemical pathways in MO3.13 cells treated with cuprizone (10μM, 48h, A), haloperidol (B), clozapine (C), cannabidiol (D) and benztropine (E), colored by p-value, created in Metascape. Colored lines represent p-values, which range between 10-20 to 10-2.
